# Hemorrhage of brain metastasis from non-small cell lung cancer post gefitinib therapy: two case reports and review of the literature

**DOI:** 10.1186/1471-2407-10-49

**Published:** 2010-02-21

**Authors:** Dan-Fang Yan, Sen-Xiang Yan, Jing-Song Yang, Yi-Xiang J Wang, Xiao-Li Sun, Xin-Biao Liao, Jun-Qing Liu

**Affiliations:** 1 Department of Radiation Oncology, the First Affiliated Hospital, College of Medicine, Zhejiang University, Hangzhou, Zhejiang 310003, PR China; 2 Departments of Diagnostic Radiology and Organ Imaging, the Chinese University of Hong Kong, Prince of Wales Hospital, Shatin, Hong Kong, PR China

## Abstract

**Background:**

Gefitinib is one of the small molecule inhibitors of epidermal growth factor receptor tyrosine kinase (EGFR TKIs). Clinical trials have demonstrated it is effective for treatment of a subset of patients with advanced non-small cell lung cancer (NSCLC). Gefitinib has been generally considered to be a relatively safe agent. Besides a small proportion of fatal interstitial pneumonia, the common adverse drug reactions of gefitinib include diarrhea and skin rash, which are generally mild and reversible. Herein, we report the first two cases of brain metastasis hemorrhage that might be involved with the use of gefitinib.

**Case presentation:**

Two patients with brain metastasis from NSCLC developed brain hemorrhage after gefitinib therapy. The hemorrhage in one case occurred one month after gefitinib combined with whole brain radiation therapy (WBRT), and in the another case hemorrhage developed slowly within brain metastases eight months post gefitinib monotherapy for diffuse pulmonary metastasis from a lung cancer undergone surgical removal previously.

**Conclusion:**

We speculate brain hemorrhage could be one of the adverse drug reactions of gefitinib treatment for NSCLC and suggest clinicians be aware of this possible rare entity. More data are needed to confirm our findings, especially when gefitinib is used in the settings of brain metastases from NSCLC or other origins.

## Background

Gefitinib is one of the inhibitors of epidermal growth factor receptor tyrosine kinase (EGFR TKIs), designed to offer targeted therapies for a variety of solid tumors including the lung cancer [1]. Clinical trials have demonstrated that gefitinib is effective or non-inferior to chemotherapy in the treatment of a subset of patients with advanced non-small cell lung cancer (NSCLC) [2-4]. Gefitinib has also been regarded as a relatively safe agent, with the most common adverse drug reactions being diarrhea and skin rash, which are generally mild in nature and reversible [5,6]. Here we describe two cases of brain metastasis from NSCLC who developed brain hemorrhage post gefitinib therapy. To our knowledge, these are the first reported cases of brain hemorrhage that might be involved in the use of gefitinib. Recently, a few hemorrhagic events in other parts of the body have also been reported after gefitinib administration [7-9]. Thus, we speculate brain hemorrhage could be one possible adverse drug reaction of gefitinib treatment for NSCLC.

## Case presentation

### Case one

**Figure 1:**
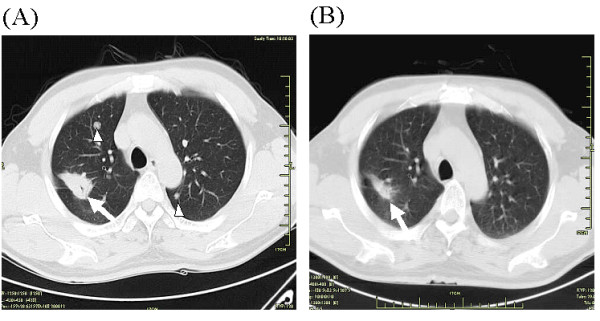
**Case 1**. **A**: Prior to gefitinib therapy, chest CT scan shows a primary lesion (arrow) in the upper lobe of right lung and multiple pulmonary metastatic nodules (arrowheads) in both lungs. **B**: One month later with gefitinib therapy, chest CT scan shows significant shrinkage of the primary lesion (arrow) as well as marked absorption of metastatic nodules.

**Figure 2:**
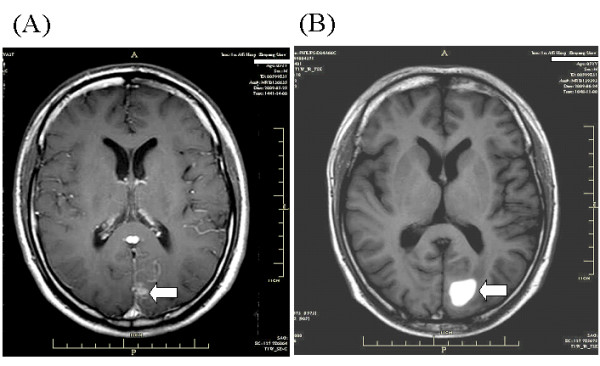
**Case 1**. **A**: Contrast-enhanced T1-weighted brain MRI shows multiple metastatic lesions, with the largest one (arrow) in the left occipital lobe. **B**: One month later with gefitinib therapy together with two weeks' WBRT, T1-weighted MRI demonstrates a subacute hematoma (arrow) in the metastatic lesion.

A 52-year-old male, who was an ex-smoker with a smoking history of ten years, was found a solitary pulmonary nodule (SPN) in the upper lobe of right lung by CT scans 6 years ago. He refused surgery or any invasive procedures to the nodule. Chest CT scan was performed every six months for follow-up. In May 2009 CT demonstrated the SPN increased in size with multiple lung and ribs metastasis (Fig. 1A). Brain MRI showed multiple metastatic lesions with the largest one in the left occipital lobe (Fig. 2A). CT-guided percutaneous needle biopsy of the pulmonary lesion proved adenocarcinoma. After refusing chemotherapy with toxic agents, gefitinib (AstraZeneca, UK) was given at a daily dose of 250 mg as the first-line treatment for NSCLC combined concurrently with whole brain irradiation (WBRT) for the metastatic brain tumors. WBRT was performed to a total dose of 30 Gy with a fraction size of 3 Gy over 2 weeks. During the course of treatment, mild skin rash and nausea and vomiting developed but were well tolerated. One month later, chest CT showed significant shrinkage of the primary lesion and marked absorption of pulmonary metastases (Fig. 1B). Meanwhile, the patient began to feel recurrent headache and nausea, and brain MRI demonstrated a left occipital lobe mass that was consistent with a subacute hematoma (Fig. 2B). The platelet counts and prothrombin time and activated partial thromboplastin time were within normal ranges. The patient had no history of diabetes or hypertension or coagulation disorders. During hospitalization the patient had no history of trauma. Except for mild to moderate headache and nausea, the patient complained of no other discomfort such as impaired orientation to person and place, hemidysesthesia or hemiopia. The patient was discharged from hospital after one week's supportive treatment with mannitol and methylprednisolone.

### Case two

**Figure 3:**
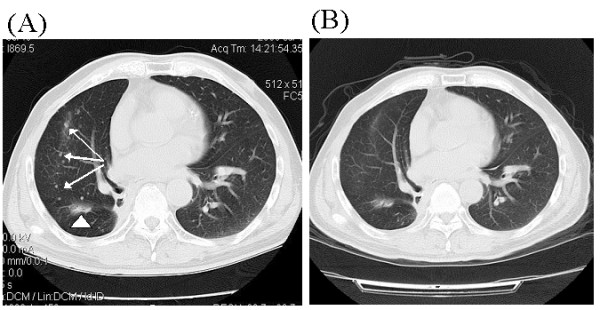
**Case 2**. **A**: Chest CT scans shows pulmonary metastatic dissemination (arrows) from a previously operated NSCLC in the right lung. A patchy shadow (arrowhead) representing radiation-induced fibrosis is also observed. **B**: About 2 month later with gefitinib therapy, chest CT scan shows significant absorption of the metastatic lesions.

**Figure 4:**
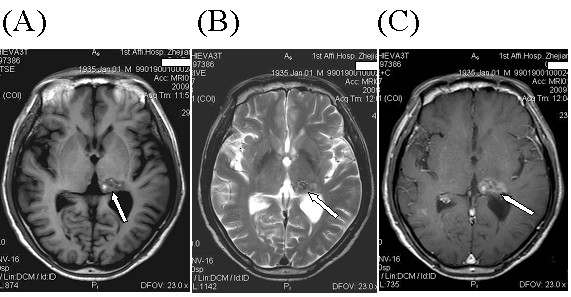
**Case 2**. **A**: T1-weighted brain MRI shows a metastatic lesion of heterogeneous signal intensities in the left thalamus (arrow). Components of hyper-signal intensities represent subacute hemorrhage. **B**: This lesion is also of heterogeneous signal intensities on T2-weighted images, with components of hypointense hemosiderins representing chronic hemorrhage (arrow). **C**: Contrast-enhanced MRI shows inhomogeneous enhancement of the lesion (arrow).

A 75-year-old male, an ex-smoker, was diagnosed with right lower lobe lung cancer in July 2007, and underwent operation after routine staging procedures. The pathology was adenocarcinoma with positive margins and ipsilateral hilar and mediastinal lymph node metastasis (Stage pT3N2M0). Postoperative adjuvant chemotherapy and thoracic radiotherapy (59 Gy/32 fractions) were administered. The patient was well tolerant to these therapies and without evidence of illness until July 2008, when CT scans demonstrated diffuse pulmonary metastatic dissemination (Fig. 3A) and a metastasis in right hepatic lobe. The patient was given gefitinib at a daily dose of 250 mg. CT scans about 2 months later showed near-CR (Complete Response) of the pulmonary lesions (Fig. 3B). After five months' medication of gefitinib, the patient developed severe bilateral paronychia. This patient had an operation of nail arrachement in November 2008. In March 2009, the patient developed right limb numbness and unstable walking. Brain MRI demonstrated a metastatic tumor in the left thalamus with intratumoral hemorrhage (Fig. 4). The platelet counting of the patient was 143,000/mm^3 ^and there were no other underlying disorders that might be related to the brain hemorrhage. WBRT was recommended but the patient preferred watchful waiting. On 5th June 2009, more metastatic lesions with evidence of bleeding were found on brain MRI and subsequently WBRT (30 Gy/15 fractions) was administered to the patient. The patient is now still alive with mild right hemiparesis.

## Discussion

Epidermal growth factor receptor (EGFR) is a member of the HER tyrosine kinase growth factor receptor family that signals cellular differentiation, proliferation, invasion, metastasis, and survival. It is expressed in a variety of human cancers including NSCLC (40%-80%), colorectal (25%-77%), pancreatic (30%-50%), breast (15%-30%), ovarian (35%-70%), prostate (40%) and gastric (33%) cancers [1]. Among the above-mentioned cancers, NSCLC was one of the most frequently studied objects of EGFR-targeted therapy, as evidenced by such clinical trials as INTEREST, ISEL (for gefitinib), and BR21 and SATURN (for erlotinib, another EGFR-TKI) [2,4,10,11].

Gefitinib (ZD1839, Iressa), a selective inhibitor of EGFR tyrosine kinase (EGFR-TKI) which is competitive with the combination of EGFR tyrosine kinase, acts through blocking the signal transduction pathway of epithelial growth factor and thereby inhibiting the proliferation and metastasis, and, promoting the apoptosis of tumor cells [1]. The orally administered gefitinib has achieved a great effect since its approval by FDA in 2003, for it has less toxicity compared with traditional cytotoxic chemotherapy. In addition to an extremely small number of fatal interstitial pneumonia reported [12], the common adverse drug reactions of gefitinib are diarrhea, skin rash, dry skin, nausea and vomiting. On most occasions these reactions are mild and reversible. Other less common adverse effects include pruritus, anorexia, asthenia, and weight loss [13]. For the above reasons, gefitinib has been used as the second or third line therapy for advanced and metastatic NSCLC, or even evaluated as the first line therapy (IPASS trial) for a subset of patients with NSCLC [14]. Our first case was administered gefitinib without prior cytotoxic therapies.

Targeted therapies also aim at other signal transduction pathways like vascular endothelium growth factor (VEGF). It was reported that anti-angiogenic therapy (AAT) had led to serious hemorrhagic events in NSCLC, particularly in those of squamous cell origin [15]. An early phase I trial of bevacizumab, an anti-VEGF monoclonal antibody, also detected a case of intracranial hemorrhage from a choriocarcinoma brain metastasis. However, a recent phase II trial, AVF3752g, showed safety of bevacizumab in patients with NSCLC and previously treated brain metastases [16]. On the other hand, anti-EGFR therapy has seldom been warned against increased risks of bleeding, except a few case reports of anecdotal experience.

There has been a case report in which severe alveolar hemorrhage occurred after four weeks' gefitinib therapy in a 56-year-old man of NSCLC in Japan [9]. Recently, another two reports on bilateral subdural hemorrhage (SDH) after oral gefitinib administration have been published [7,8]. In one, a 75-year-old woman was diagnosed as stage IV NSCLC and was introduced to take gefitinib at a daily dose of 250 mg. About 7 months later, the patient gradually developed headache and weakness, and CT demonstrated bilateral SDH another 2 months later. Although there was no obvious evidence of CNS metastasis, the authors still thought that bilateral SDH might had resulted from obstruction of dural vessels by latent dural metastasis and was also suggested as a possible adverse event of gefitinib therapy. Notably, the above-described two cases developed hemorrhagic events without CNS metastasis. Huang et al [8] reported another case where a 57-year-old male developed bilateral SDH after WBRT combined with EGFR-TKIs for NSCLC with brain metastases. Gefitinib was replaced with erlotinib on the 5th day after WBRT. During a follow-up period with erlotinib alone after completing WBRT, the patient developed bilateral SDH. The authors did not make a clear explanation for the complication. Apart from the above reports of hemorrhagic events that might be related to gefitinib in NSCLC, there was also a recent phase III study showing gefitinib dose-dependently increased tumor hemorrhage-type events in recurrent squamous cell carcinoma of the head and neck [17].

In the current report, two patients developed brain metastasis hemorrhage after taking gefitinib. Though in our first case brain tumor hemorrhage developed one month after a combination of gefitinib and WBRT, it is still possible that gefitinib played a role. There were no comorbiditis such as thrombocytopenia, coagulation abnormalities or other underlying cerebrovascular diseases or head trauma, and our own experience and reports from other authors suggested that WBRT alone was unlikely to be the cause of tumoral hemorrhage of brain metastases, but rather could decrease the hemorrhagic events through blunting angiogenesis and normalizing tumor vasculature [18,19]. As considerable studies have demonstrated gefitinib to be a radiation sensitizer in the treatment of a variety of tumors including NSCLC, head and neck, breast, and colorectal cancers [20,21], we think EGFR-targeted therapy may have strengthened the role of radiation-induced vascular occlusion and subsequent post-ischemic hemorrhage. Furthermore, gefitinib in combination with WBRT might lead to rapid shrinkage of brain metastasis and avascular necrosis, and induce reconstruction of microvasculature and vascular abnormality, subsequently leading to tumoral bleeding. This is especially true for those with high expression or mutations of EGFR. Unfortunately, our two cases have not been performed that kind of test, though patients now in our institute are strongly encouraged to take such test before taking gefitinib or other TKIs.

In the second case, hemorrhagic brain metastasis was found 8 months after gefitinib monotherapy. Before that, the patients also experienced severe paronychia and underwent a nail arrachement three months after gefitinib administration. MRI showed evidence of both subacute (hyperintensities on T1-weighted image) and chronic (hypointensities on T2-weighted image) components of bleeding, with no obvious perilesional edema, which might accounted for the mild symptoms and signs of the patients. For the above reasons, the patient was not immediately administered WBRT until some new hemorrhagic metastases were detected in other parts of the brain. As reported in the literature, the incidence of spontaneous intracranial hemorrhage from NSCLC with brain metastasis seemed to be very low (about 1.2%), though higher than those without CNS metastasis [22,23]. Furthermore, recent studies on EGFR inhibition with gefitinib also showed its influence on angiogenesis [24]. Hence, though whether the brain metastasis hemorrhages were just coincidence or as a result of gefitinib therapy remain to be further confirmed, it seems possible that gefitinib might be involved in the brain hemorrhage in our two cases. Interestingly, there is a study on treatment of brain metastasis from NSCLC with WBRT and gefitinib which showed acute side effects were generally well tolerated, and no hemorrhagic events were mentioned [6], our experience suggest that caution should be taken when gefitinib is used in combination with WBRT.

## Conclusion

In summary, from our cases and others reported in the literature, we speculate that brain metastasis hemorrhage could be a possible adverse drug reaction of gefitinib for treatment of NSCLC. With increasing use of gefitinib, especially in those patients with EGFR over-expression or mutations, it is reasonable to suggest that clinicians be highly cautious about this possible complication.

## Consent

Written informed consent was obtained from the patient for publication of this case report and any accompanying images. A copy of the written consent is available for review by the Editor-in-Chief of this journal.

## Competing interests

The authors declare that they have no competing interests.

## Authors' contributions

DFY and YXJW analyzed the data and wrote the manuscript. JSY, XLS, XBL and JQL made substantial contributions in data acquisition and data interpretation. SXY participated in study design and coordination. All authors read and approved the final manuscript.

## Pre-publication history

The pre-publication history for this paper can be accessed here:

http://www.biomedcentral.com/1471-2407/10/49/prepub
